# Confirmatory Clinical Validation of a Serum-Based Biomarker Signature for Detection of Early-Stage Pancreatic Ductal Adenocarcinoma

**DOI:** 10.3390/curroncol32110638

**Published:** 2025-11-13

**Authors:** Patricio M. Polanco, Tamas Gonda, Erkut Borazanci, Evan S. Glazer, Jose G. Trevino, George DeMuth, Lisa Ford, Thomas King, Norma A. Palma, Randall E. Brand

**Affiliations:** 1 Division of Surgical Oncology, University of Texas Southwestern Medical Center, Dallas, TX 75390, USA; 2 Division of Gastroenterology & Hepatology, New York University, New York, NY 10016, USA; 3 Department of Oncology, HonorHealth Research Institute, Scottsdale, AZ 10510, USA; 4 Division of Surgical Oncology, University of Tennessee Health Science Center, Memphis, TN 38163, USA; eglazer@uthsc.edu; 5 Division of Surgical Oncology, School of Medicine and Surgeons, Virginia Commonwealth University, Richmond, VA 23298, USA; 6 Stat One, LLC, Morrisville, NC 27560, USA; 7 Immunovia, Inc., Durham, NC 27713, USAthomas.king@immunovia.com (T.K.); 8 Division of Gastroenterology, Hepatology and Nutrition, University of Pittsburgh Medical Center, Pittsburgh, PA 15261, USA

**Keywords:** pancreatic ductal adenocarcinoma, early detection, biomarkers, high-risk, pathogenic germline mutation, mucinous pancreatic cyst

## Abstract

Early detection of pancreatic ductal adenocarcinoma (PDAC) could extend patient survival, and biomarkers to facilitate this are urgently needed. Here, we performed a second independent validation of PancreaSure, a 5-plex serum biomarker signature to detect early-stage PDAC in high-risk individuals. In contrast to the first validation, this study’s cohort was preemptively balanced for age and sex and only included samples stored for fewer than 5 years. The primary endpoint was to measure test sensitivity against the performance target of 65%. Measuring specificity against the performance target of 90% and comparing test performance to that of carbohydrate antigen 19-9 (CA 19-9) alone were secondary endpoints. Signature analytes were retrospectively measured in serum from a blinded independent cohort of Stage I and II PDAC cases and high-risk controls. A predictive signal for PDAC was generated from a predefined cutoff established in a previous model development study. PancreaSure distinguished early-stage PDAC from controls with 76.5% sensitivity (95% CI, 67.7–83.9), significantly higher than the performance target (*p* = 0.005). PancreaSure achieved 87.8% specificity (95% CI, 83.9–91.4), similar to the performance goal, and significantly outperformed sensitivity of CA 19-9 alone (*p* = 0.02). These results confirm that PancreaSure performs well at detecting early-stage PDAC in high-risk individuals.

## Simple Summary

Pancreatic ductal adenocarcinoma (PDAC) has one of the lowest 5-year survival rates of all malignancies, mainly because most cases are diagnosed at late stages. Diagnosing PDAC during early stages (Stage I and II) significantly improves patient survival. To aid in the early detection of PDAC, we developed and clinically validated a serum biomarker signature to distinguish early-stage PDAC in those at high risk of developing the disease. In this study, we performed a second validation of the signature in an independent group of people who were either recently diagnosed with Stage I or II PDAC or were at higher risk of developing PDAC due to a gene mutation, strong family history of PDAC, and/or a mucinous pancreatic cyst. The biomarker signature detected PDAC with high sensitivity and specificity, showing that it has potential to aid in early detection of this malignancy.

## 1. Introduction

Pancreatic cancer is the third leading cause of cancer death in the United States and one of the few cancers that is becoming more prevalent worldwide [1,2,3]. This malignancy tends to remain asymptomatic until after it has metastasized and therefore is often diagnosed at late stages when surgical resection is no longer an option [4]. Pancreatic ductal adenocarcinoma (PDAC), which accounts for 90% of all pancreatic cancers [5], currently has a 5-year survival rate of 3.1% when diagnosed at Stage IV [6], but this rate improves to 44% when diagnosis occurs prior to metastasis (early-stage PDAC) and to 83% when diagnosed at Stage IA [6,7]. These statistics highlight the significant impact that early detection can have on patient outcomes and underscore the dire need to increase early-stage diagnoses if patient survival rates are to be extended.

To facilitate early detection, current guidelines recommend yearly imaging surveillance for those at high risk of developing PDAC due to having a pathogenic germline variant (PGV) in a PDAC susceptibility gene, a strong family history of PDAC (typically > 2 affected relatives in first-degree relation (FDR) to each other, one of whom is a FDR to the individual in surveillance), or a mucinous pancreatic cystic neoplasm [8,9,10]. Even though surveillance has been shown to increase early-stage diagnosis and prolong survival [11,12], late-stage PDAC and interval tumors still occur in surveillance populations [13,14,15], demonstrating the limitations of imaging and the need for complementary biomarker-based surveillance tests [16,17].

Despite decades of promising biomarker discovery and preclinical findings [18,19,20], reliable biomarkers to aid in PDAC diagnosis remain unavailable, primarily due to the rarity of samples that capture pre-diagnosis, early-stage disease, and high-risk physiologies [2,17,21]. Carbohydrate antigen 19-9 (CA 19-9), the only biomarker approved for use in PDAC patients, is recommended for disease and treatment monitoring, but not for diagnosis due to its low sensitivity and specificity (60–68%) [22,23]. However, CA 19-9 has been shown to improve diagnostic success of PDAC when included in panels of other biomarker candidates [24,25,26,27,28,29,30,31].

PancreaSure is a novel serum biomarker signature comprising tissue inhibitor of metalloproteinase 1 (TIMP1), intercellular adhesion molecule 1 (ICAM1), cathepsin D (CTSD), thrombospondin 1 (TSP1/THBS1), and CA 19-9 that was designed to detect early-stage PDAC [32]. In our previous clinical validation study [33], PancreaSure distinguished Stage I and II PDAC (*n* = 202) from high-risk controls (*n* = 864) with 78.2% sensitivity and 93.5% specificity. Due to the retrospective nature of that study and a female predominance typically seen in PDAC surveillance populations, the cohort demonstrated disparities in age and sex matching and included samples that had been in −80 °C storage for up to 10 years. The test performed significantly worse in samples that had been in storage for more than 5 years. The first validation also lacked high-risk controls that reported mucinous pancreatic cysts but no genetic/familial susceptibility to PDAC. The goal of this study was to address these limitations by validating PancreaSure in its intended use population on a cohort that: (1) was age- and sex-balanced, (2) only included samples stored for fewer than 5 years, (3) included high-risk controls with pancreatic cysts but no genetic/familial susceptibility, and (4) was completely different from the cohort used in the first validation study.

## 2. Materials and Methods

### 2.1. Study Endpoints

The primary endpoint was to evaluate the sensitivity of PancreaSure in an age- and sex-balanced cohort of early-stage PDAC and high-risk control samples less than 5 years old against the pre-defined success criterion of 65%. During assay development the observed sensitivity of CA 19-9 alone was 65%, which established this metric as a comparator for validation. Evaluating specificity was a secondary endpoint because reductions made to balance age and sex resulted in a relatively low number of control samples. The success criterion for specificity was 90% because the test was optimized towards 98% specificity during development [32] and demonstrated 93.5% specificity in the first validation study [32]. Comparing PancreaSure’s performance to CA 19-9 alone was also a secondary endpoint. Exploratory study endpoints included performance evaluations in cohort sub-populations. We note that PancreaSure’s threshold for determining a Positive or Negative test result was not recalibrated for this study because the primary aim was to test its performance in the same clinical populations (high-risk surveillance and early-stage PDAC) in which the assay was developed and validated [32,33]. The cohort in this study intentionally contained the same patient groups as those used in the 2 prior studies to enable an accurate evaluation of test performance in its intended use population.

### 2.2. Study Design and Population

This retrospective study was conducted according to the approved study protocol (available from the corresponding author upon reasonable request). Briefly, banked serum samples were obtained from 6 institutions across the United States (Supplemental Table S1). All institutions had Institutional Review Board (IRB) approval for collection of blood samples and clinical data. All study participants provided written informed consent to the respective institutions that permitted use of their samples in this study. Samples were selected for study according to Supplemental Figure S1 and then deidentified and assigned a unique clinical identifier to conceal participant data and disease status from laboratory personnel. The study population comprised (1) treatment naïve patients recently diagnosed with Stage I or II PDAC based on imaging and (2) controls who were at increased risk of developing PDAC due to the presence of a pathogenic germline variant (PGV) in a PDAC susceptibility gene, strong family history of PDAC (≥2 affected relatives in first-degree relation (FDR) to each other, one of whom is a FDR to the individual in surveillance), or at least one mucinous pancreatic cyst between 1.0 and 3.0 cm in diameter (Table 1). Cases with recurrent PDAC or any active non-PDAC malignancy other than basal or squamous cell carcinoma of the skin 3 or more years prior to sample collection were excluded. Cases that reported an active non-PDAC cancer more than 3 years before sample collection were included. Controls with either biliary obstruction secondary to gallstones, chronic pancreatitis, or cystic pancreatic lesions greater than 3.0 cm in diameter were excluded. Samples were preemptively balanced for age and sex. All samples chosen for analysis had been stored at −80 °C for fewer than 5 years.

### 2.3. Biomarker Measurements

TIMP1, ICAM1, CTSD, and THBS1 were measured in serum using analytically validated enzyme-linked immunosorbent assays (ELISAs) as previously described (Pescatore et al., unpublished data, submitted September 2025). CA 19-9 was measured using a COBAS 8000 modular analyzer (Roche Diagnostics, Rotkreuz, Switzerland) according to the manufacturer’s instructions. All measured analyte concentrations were logarithmically transformed, multiplied by an established coefficient for each analyte, and the sum of results for each sample was assigned a Positive or Negative prediction based on a predefined cutoff (threshold) determined in the assay development study [32].

### 2.4. Statistical Analyses

#### 2.4.1. Test Performance

115 cases and 270 controls were chosen to provide 82.8% power for a one-sided 0.025 exact binomial test of sensitivity based on the performance goal of 65%, thus allowing the primary study endpoint to be achieved. The relationship between individual biomarkers and age, sex, and PDAC status and stage was analyzed using Mann–Whitney U tests. When used as a standalone biomarker, CA 19-9 ≥ 37 U/mL was considered PDAC positive and CA 19-9 < 37 U/mL was considered PDAC negative. Diagnostic sensitivities and specificities in the full cohort and in relevant sub-populations were compared using either McNemar or two-proportion Z-tests. Univariate biomarker analyses were compared using two-tailed student’s t-tests. For all tests, *p*-values < 0.05 were considered significant.

#### 2.4.2. Exploratory Analyses

Test performance was evaluated in those ≥65 years of age, and in individuals diagnosed with diabetes or pancreatic cyst(s) only. Comparisons were also made between Stage I and II PDAC and between PDAC cases that developed in persons with and without genetic/familial susceptibility.

#### 2.4.3. Analysis Software

All statistical analyses were performed using either SAS 9.4 TS1M8 (9.4 M8) or R, version 4.3.1 [34].

## 3. Results

### 3.1. Participant Characteristics

Serum samples from 385 study participants were retrospectively analyzed in a blinded manner (*n* = 115 cases, *n* = 270 controls) (Table 2). The median age of all study participants was 67 years (SD ± 8.8 years), and age was balanced between cases (68 years, SD ± 8.8 years) and controls (67 years, SD ± 8.7 years) (*p* = 0.061). There was statistically insignificant (*p* = 0.151) female predominance in the control group (56.7%) and in the full cohort (54.3%). The total number of cases were balanced between Stage I (48.7%) and Stage II (51.3%) malignancies (*p* = 0.704).

Based on NCCN guidelines [9], 100% of controls were high-risk due to genetic/familial susceptibility and/or the presence of at least one mucinous pancreatic cyst. 80.7% of controls had a PGV and/or family history of PDAC. 28.1% of controls had both a PGV/family history and mucinous cyst. 19.2% of controls reported a cyst only (Supplemental Figure S2). 73% of cases developed in persons who were not at increased risk of PDAC. 21.7% of cases reported a PGV or PDAC family history. 5.2% of cases reported genetic/familial susceptibility and a cyst. 8.6% of cases reported a cyst only (Supplemental Figure S2).

### 3.2. Individual Analyte Expression

We characterized the expression of individual analytes relative to age, sex, PDAC status, and collection site. In agreement with our previous studies, TIMP1, ICAM1, CTSD, and CA 19-9 were significantly different between PDAC cases and controls (*p* < 0.001). In contrast to our previous findings, THBS1 was not significantly different in this cohort (*p* = 0.291) (Supplemental Table S2). Age was not associated with the levels of ICAM1, CTSD, THBS1, or CA 19-9. TIMP1 was significantly associated with age in controls (*p* = 0.008) (Supplemental Figure S3). CA 19-9 levels were significantly higher in male cases than female cases (*p* = 0.018) (Supplemental Table S3), yet no associations between sex and the remaining analytes were observed. Analyte levels varied based on sample collection site (Supplemental Table S4).

### 3.3. Clinical Performance

#### 3.3.1. Overall Performance

PancreaSure detected Stage I and II PDAC with 76.5% sensitivity (95% CI, 67.7–83.9), significantly outperforming the performance goal of 65% (*p* = 0.005) and achieving the primary study endpoint. The test distinguished PDAC with 87.8% specificity (95% CI, 83.3–91.4), just below the performance goal of 90% and achieved significantly higher sensitivity than CA 19-9 alone (68.7%, *p* = 0.02) (Table 3 and Supplemental Figure S4), to achieve 1 of the 2 secondary endpoints.

#### 3.3.2. Sub-Populations

We evaluated PancreaSure’s performance in study participants diagnosed with diabetes, those with mucinous pancreatic cysts only, and those ≥65 years of age. We also evaluated performance between Stage I and II malignancy, and in individuals who developed PDAC with and without genetic and/or familial susceptibility. The lowest performance was observed in individuals diagnosed with cysts (68.8% sensitivity and 85.3% specificity) (Table 4). Diabetics and those ≥65 years of age demonstrated sensitivities of 77.8% and 77.5%, respectively. Diabetics demonstrated the highest specificity at 90.6%. Those ≥65 years achieved 87.2% specificity. None of the performances observed in these sub-groups were significantly different from the test’s performance in the rest of the cohort. Performance was consistent between Stage I and II PDAC (*p* = 0.57) and between cases that developed in individuals with and without genetic/familial susceptibility (*p* = 0.99).

#### 3.3.3. Collection Sites

We evaluated test performance based on sample collection site. Specificities ranged from 55.6% to 93.2% and sensitivities ranged from 0% to 100% (Supplemental Table S5). The 1 site that achieved 0% sensitivity did so because the single case sample received from there was a false negative. Likewise, 1 institution achieved 55.6% specificity because 4 of the 9 control samples received from that location were false positives.

## 4. Discussion

The primary aim of this study was to perform a second validation of PancreaSure in an independent cohort of early-stage PDAC cases and high-risk controls that was preemptively balanced between age and sex, only included samples that were less than 5 years of age and included high-risk controls with pancreatic cysts but no genetic/familial susceptibility. PancreaSure detected PDAC with 76.5% sensitivity, significantly outperforming the pre-defined success criterion of 65% (*p* = 0.005). The test detected PDAC with 87.8% specificity, similar to the performance goal of 90%, and achieved significantly higher sensitivity than CA 19-9 alone (68.7%, *p* = 0.02). Test performance was consistent between Stage I and II cases, between cases that developed in individuals with and without genetic and/or familial susceptibility to PDAC, and between all sub-groups tested and the rest of the cohort.

PancreaSure is intended for patients who are undergoing PDAC surveillance due to documented PGVs, family history, and/or the presence of mucinous pancreatic cysts. It is designed to be used between imaging examinations with the goal of helping to increase early detection of interval cancers (high specificity) and, to the extent possible, extend the interval between imaging examinations (high sensitivity). The performance achieved by PancreaSure suggests that it has potential to facilitate early detection in this population.

Routine surveillance with endoscopic ultrasound (EUS), magnetic resonance imaging (MRI), or computerized tomography (CT) increases PDAC diagnosis at early stages and significantly extends survival [11,12]. However, imaging is time consuming and poses safety risks to patients and interval cancers still occur in those who undergo surveillance. PancreaSure offers an advantage by virtue of its non-invasiveness, lower time burden, and greater accessibility relative to imaging. Furthermore, for tumors <2 cm in diameter (Stage I cases), PancreaSure achieved higher sensitivity (72.4%) than that reported for CT (58–77%) [35,36]. While direct comparisons are needed to fully evaluate PancreaSure’s performance relative to imaging, these findings demonstrate its potential to be a valuable asset to early-stage detection.

The lifetime risk of PDAC in the general population is ~1.5%, and in surveillance populations approximately 1 in 200 imaging procedures results in a diagnosis [11,37]. For a disease with such low prevalence a diagnostic test with high specificity is ideal because it would limit the number of false positive test results and reduce unnecessary follow-up imaging procedures and patient anxiety. PancreaSure’s specificity of 87.8% did not meet the acceptance criterion (90%) or outperform the specificity demonstrated by CA 19-9 alone. However, its specificity was nonetheless high, suggesting that PancreaSure’s potential to impact clinical outcomes in surveillance populations should not be discounted.

This study had many unique strengths. One, our study’s control group contained only high-risk individuals. Historically, this group has been underrepresented in biomarker discovery and validation studies and is cited as a primary reason why biomarkers to aid in PDAC diagnosis have not been successful in clinical trials [38,39]. Two, by preemptively balancing this study’s cohort for age and sex, limiting analysis to samples less than 5 years of age, and including a sub-population of high-risk controls with mucinous pancreatic cysts but no genetic/familial susceptibility, we addressed the limitations of the first validation study [33] and still report high test performance in its intended use population. Three, our study only included PDAC cases with Stage I and II disease. Given that biomarker performance is typically lowest in early-stage disease, high performance in Stage I and II PDAC is notable. Furthermore, biomarkers that perform well in Stage I and II combined, but poorly in Stage I alone, are unlikely to impact patient survival because many individuals diagnosed at Stage II succumb to their cancer within 5 years [2,7]. For this reason, the consistent performance demonstrated between Stage I cases alone and Stage II cases alone is especially noteworthy.

Although these findings are promising, we acknowledge that our study had limitations. The current study’s cohort was smaller than the cohorts used in our previous validation and model development studies [32,33], which was the result of removing samples to preemptively balance age and sex. This limitation also attests to the rarity of early-stage PDAC samples. Higher sample numbers are especially needed to thoroughly characterize PancreaSure’s performance in clinically relevant sub-populations including diabetics, those ≥65 years of age, and those with mucinous pancreatic cysts but no genetic/familial susceptibility to PDAC. We are currently addressing this gap in knowledge by analyzing pooled datasets from this study and the first validation, to provide higher statistical power for the evaluation of test performance in sub-groups. There was also the observation that THBS1 expression was not significantly different between PDAC cases and controls. THBS1 expression reportedly increases in many cancer types and is often associated with poor prognosis [40]. Yet, in the first clinical validation we observed significantly lower THBS1 expression in PDAC cases relative to controls [33], and this phenomenon has been reported in pancreatic cancer patients up to 24 months prior to diagnosis [27,41]. THBS1 plays multiple roles in promoting malignancy, including by suppressing angiogenesis to promote dormancy and allowing tumor cells to escape detection by the immune system [42]. Here, the lack of a significant difference in THBS1 expression between cases and controls may be the result of having a smaller cohort in this study or disparities in biomarker expression between institutions. However, the similar test performances observed in this study and the first validation suggest that discrepancies in THBS1 expression between the two cohorts had minimal impact on overall test results. This observation is not surprising given that the assay was not designed to show statistical significance at the univariate level, but rather, at the level of the full signature. Nonetheless we acknowledge the scientific value in evaluating the individual contribution made by each analyte to the test’s result and intend to explore this in future studies. Another limitation was the imbalance between samples received from each collection site, which constrained institution-specific analyses. Some institutions contributed only 1 or 2 samples per study group, which resulted in low sensitivity and specificity for those sites. Balanced site-level cohorts will be addressed in future studies. Another study limitation was the study’s retrospective design, which inherently introduces the potential for selection bias, as patient inclusion may have been influenced by factors not accounted for in the analysis. Retrospective data collection is subject to incomplete or inconsistent documentation, which may lead to missing or inaccurate clinical information. Finally, since PancreaSure was designed to detect Stage I and II PDAC, and to date has only been tested in high-risk controls, its performance in Stage III and IV PDAC and in healthy controls remain untested. However, since biomarker test characteristics typically improve with increasing tumor burden, we expect PancreaSure to perform well in both advanced-stage PDAC and in the test’s intended use population compared to healthy individuals. Validations to assess test performance in these populations are currently underway.

Altogether, this study confirms that PancreaSure detects early-stage PDAC with high sensitivity and specificity in a cohort independent from the ones in which it was developed and first validated, providing additional evidence of its potential to improve early-stage detection in surveillance populations and relevant sub-groups.

## Figures and Tables

**Table 1:** Study inclusion/exclusion criteria.

Inclusion Criteria		Exclusion Criteria	
Able to provide written informed consent. ≥45 years of age.		Major surgery or significant trauma within 12 weeks prior to blood sample collection.	
Cases	Controls	Cases	Controls
Treatment-naïve, pathologically confirmed PDAC Stage I or Stage II. Sporadic or familial/genetic PDAC.	Individuals at high risk for PDAC because of their familial and/or genetic history per NCCN Guidelines [9]. Individuals at high risk for PDAC because of pancreatic cysts between 1.0 and 3.0 cm in diameter. Similar demographics (age/gender) to PDAC patients.	Prior treatment for PDAC (e.g., prior resection, radiotherapy, or chemotherapy). Current immunosuppressive or chemotherapy. Active non-PDAC malignancies or active co-morbid malignancies other than basal or squamous cell carcinoma of the skin ≤3 years prior to sample collection.	Biliary obstruction secondary to gallstones. Prior diagnosis/imaging evidence of chronic pancreatitis. Cystic pancreatic lesions >3.0 cm in diameter or showing worrisome features according to Fukuoka Consensus Guidelines.

**Table 2:** Study participant characteristics.

Variable		All (*n* = 385)	Cases (*n* = 115)	Controls (*n* = 270)	*p*-Values ^†^
Age (years), Mean (SD) Range		67 (8.8) 45–88	68 (8.8) 48–88	67 (8.7) 45–88	0.061
Sex	Male	176 (45.7%)	59 (51.3%)	117 (43.3%)	0.151
	Female	209 (54.3%)	56 (48.7%)	153 (56.7%)	
Family history of PDAC	Yes	205 (53.2%)	19 (16.5%)	186 (68.9%)	<0.001
	No	180 (46.8%)	96 (83.5%)	84 (31.1%)	
High Risk Mutation in a PDAC susceptibility gene *	Yes	59 (15.3%)	7 (6.1%)	52 (19.3%)	0.005
	No	326 (84.7%)	108 (93.9%)	218 (80.7%)	
Combined Genetic/Familial High-Risk	Yes	249 (64.7%)	31 (27%)	218 (80.7%)	<0.001
	No	136 (35.3%)	84 (73%)	52 (19.3%)	
Mucinous Pancreatic Cyst(s)	Yes	145 (37.7%)	16 (13.9%)	129 (47.8%)	<0.001
	No	240 (62.3%)	99 (86.1%)	141 (52.2%)	
CA 19-9	37 U/mL	108 (28%)	79 (68.6%)	29 (10.7%)	<0.001
	<37 U/mL	277 (71.9%)	36 (31.3%)	241 (89.2%)	
Diabetes	Yes	77 (20%)	45 (39.1%)	32 (11.9%)	<0.001
	No	308 (80%)	70 (60.9%)	238 (88.1%)	
PDAC Stage	Stage 1	56 (14.5%)	56 (48.7%)	N/A	0.704
	Stage 2	59 (15.3%)	59 (51.3%)	N/A	

* *ATM*, *APC*, *BRCA1*, *BRCA2*, *BRCA1/2*, *PALB2*, *CDKN2A*, *STK11*, *MSH2*, *MSH6*, or *MLH1*. **^†^** Comparisons between cases and controls were assessed using chi-square or Fisher’s exact tests for categorical variables and t-tests for continuous variables. All *p*-values show comparisons between cases and controls except for PDAC stage which compares the distribution between Stage I and II cases. N/A = not applicable.

**Table 3:** Performance comparisons between PancreaSure and CA 19-9 alone.

Model	Sensitivity (n/N)	Sensitivity *p*-Value	Specificity (n/N)	Specificity *p*-Value
PancreaSure	76.5% (88/115)	0.02	87.8% (237/270)	0.58
CA 19-9	68.7% (79/115)		89.3% (241/270)	

**Table 4:** Test performance in cohort sub-populations.

Group	Cases	Controls	Sensitivity (95% CI)	Sensitivity *p*-Value *	Specificity (95% CI)	Specificity *p*-Value *
Full cohort	115	270	76.5% (68.8–84.3)		87.8% (83.9–91.7)	
≥65 years	80	156	77.5% (56.7–87.5)	0.708	87.2% (80.9–92.0)	0.726
Diabetes	45	32	77.8% (62.9–88.8)	0.799	90.6% (75.0–98.0)	0.600
Mucinous pancreatic cysts	16	128	68.8% (41.3–89.0)	0.335	85.3% (78.0–90.9)	0.229
	**Cases**	**Controls**	**Sensitivity** **(95% CI)**	**Sensitivity *p*-value ^†^**	**Specificity** **(95% CI)**	**Specificity** ***p*-value ^†^**
Stage I PDAC	56	270	72.4% (60.9–83.9)	0.5750	87.8% (83.9–91.7)	0.99
Stage II PDAC	59	270	78.8% (67.7–89.9)		87.8% (83.9–91.7)	
Sporadic PDAC	84	270	76.2% (67.1–85.3)	0.99	87.8 (83.9–91.7)	0.99
Genetic/familial risk	31	218	77.4% (62.7–92.1)		88.1 (83.8–92.4)	

* Comparisons between subgroups and the rest of the cohort. **^†^** Comparisons between Stage I and II cancers, and between sporadic and genetic/familial cases.

## Data Availability

The original contributions presented in this study are included in the article/Supplementary Materials. Further inquiries can be directed to the corresponding author.

## Supplementary Materials

The following supporting information can be downloaded at: https://www.mdpi.com/article/10.3390/curroncol32110638/s1, Table S1: Sample collection sites; Figure S1: Method of sample selection; Figure S2: Summary of high-risk clinical and genetic/familial traits represented in the study cohort; Table S2: Analyte expression in PDAC cases and controls; Figure S3: Association between biomarker expression and age; Table S3: Analyte expression in males and females; Table S4: Analyte expression at each collection site; Figure S4: ROC curves and AUC values of PancreaSure and CA 19-9; Table S5: Test performance by collection site.

## Abbreviations

The following abbreviations are used in this manuscript:

CA 19-9	Carbohydrate antigen 19-9
CI	Confidence interval
CTSD	Cathepsin D
ICAM1	Intercellular adhesion molecule 1
THBS1	Thrombospondin 1
TIMP1	Tissue inhibitor of metalloproteinase 1
