# Supplementary material for: Confirmatory Clinical Validation of a Serum-Based Biomarker Signature for Detection of Early-Stage Pancreatic Ductal Adenocarcinoma
Source: Curr Oncol. 2025 Nov 13;32(11):638. doi: 10.3390/curroncol32110638 (PMC12651218; doi:10.3390/curroncol32110638)
Supplement: Supplementary file 1 [file curroncol-32-00638-s001.zip › Figure S2.pdf]

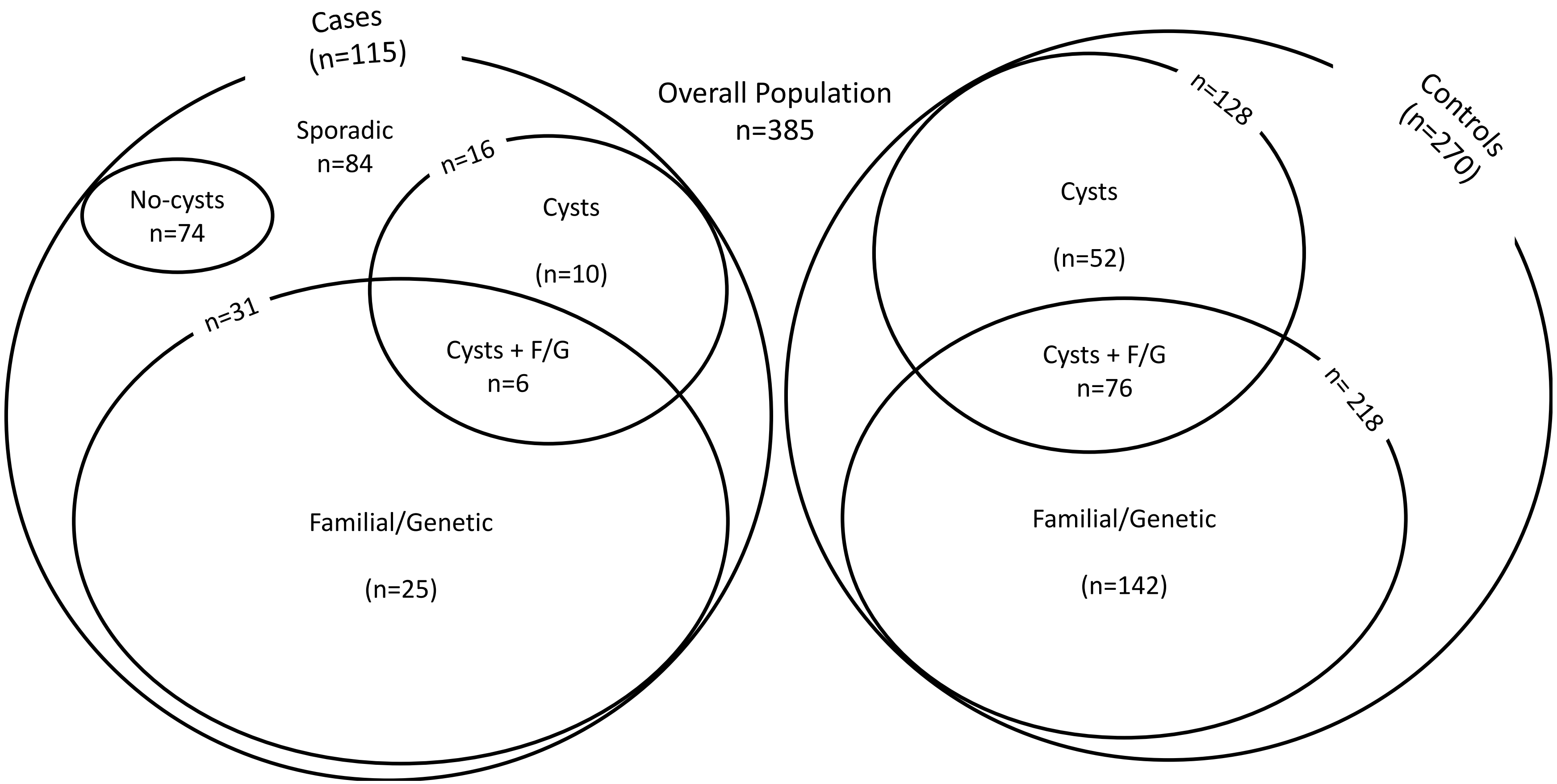

Supplemental Figure 2. Venn diagrams showing percentages of cases (**left**) and controls (**right**) that reported a PGV, PDAC family history, and/or mucinous pancreatic cyst(s).
