# Supplementary material for: Confirmatory Clinical Validation of a Serum-Based Biomarker Signature for Detection of Early-Stage Pancreatic Ductal Adenocarcinoma
Source: Curr Oncol. 2025 Nov 13;32(11):638. doi: 10.3390/curroncol32110638 (PMC12651218; doi:10.3390/curroncol32110638)
Supplement: Supplementary file 1 [file curroncol-32-00638-s001.zip › Figure S3.pdf]

A.

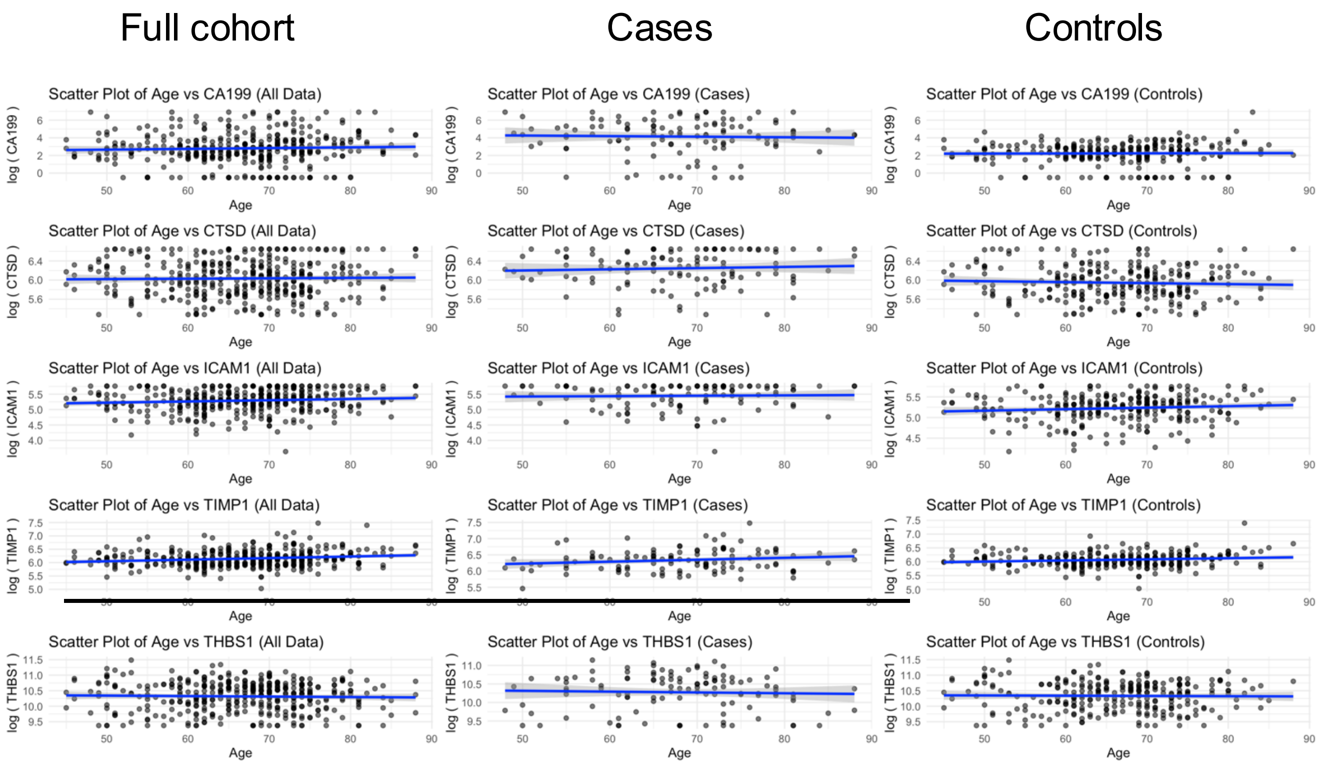

B.

Analyte	Full Cohort			Cases			Controls		
	R	R-square	p-value	R	R-square	p-value	R	R-square	p-value
TIMP1	0.178	0.0315	< 0.001	0.154	0.0238	0.0998	0.161	0.0258	0.0082
ICAM1	0.109	0.0119	0.0325	0.043	0.0018	0.6487	0.103	0.0107	0.0906
CTSD	0.026	0.0007	0.6166	0.088	0.0077	0.3512	-0.065	0.0043	0.2843
THBS1	-0.065	0.0042	0.204	-0.057	0.0033	0.5432	-0.061	0.0038	0.3147
CA199	0.058	0.0033	0.2581	-0.04	0.0016	0.6743	0.133	0.0177	0.0288

Supplemental Figure 3. Association between biomarker expression and age. (**A**) Scatterplots showing distribution of analyte expression as a function of age in the full cohort, cases, and controls. (**B**) Table showing the comparisons of age to log-transformed analyte concentrations.
