# Supplementary material for: Confirmatory Clinical Validation of a Serum-Based Biomarker Signature for Detection of Early-Stage Pancreatic Ductal Adenocarcinoma
Source: Curr Oncol. 2025 Nov 13;32(11):638. doi: 10.3390/curroncol32110638 (PMC12651218; doi:10.3390/curroncol32110638)
Supplement: Supplementary file 1 [file curroncol-32-00638-s001.zip › Figure S4.pdf]

A.

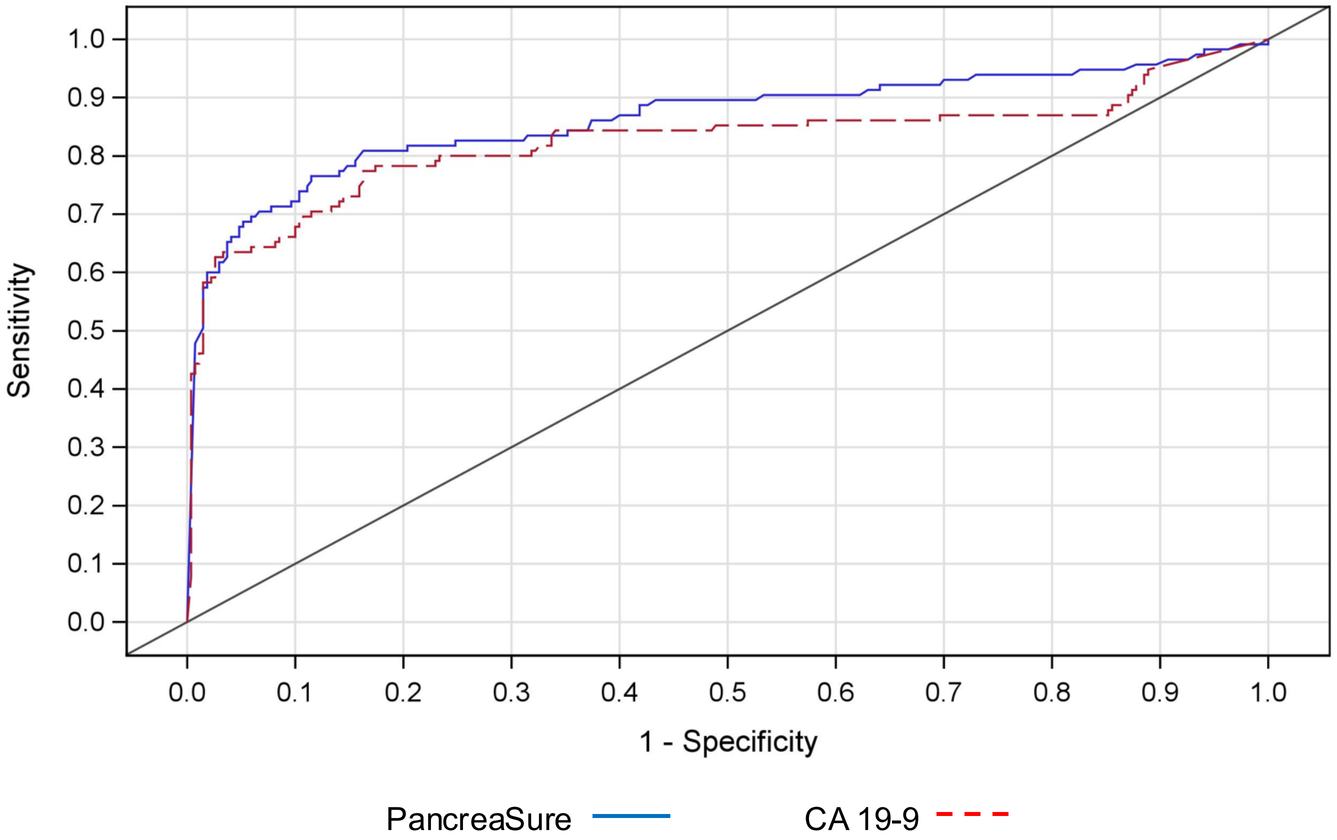

B.

		Difference in AUC (PancreaSure-CA 19-9)	
	AUC (95% CI)	Estimate	95% CI (P-value[1])
PancreaSure	0.864 (0.816-0.913)	0.039	-0.006-0.098 (0.092)
CA 19-9	0.825 (0.768-0.882)		

[1] P-value for the null hypothesis that the difference in the AUC is equal to zero.

Supplemental Figure 4. (**A**) ROC curves showing the performance of PancreaSure and CA 19-9 alone. Gray line depicts no discrimination between groups (AUC= 0.5). (**B**) Table showing AUC values.
