# Supplementary material for: Confirmatory Clinical Validation of a Serum-Based Biomarker Signature for Detection of Early-Stage Pancreatic Ductal Adenocarcinoma
Source: Curr Oncol. 2025 Nov 13;32(11):638. doi: 10.3390/curroncol32110638 (PMC12651218; doi:10.3390/curroncol32110638)
Supplement: Supplementary file 1 [file curroncol-32-00638-s001.zip › Table S1.pdf]

Supplemental Table 1. Sample collection sites.

Institution	Cases (n)	Controls (n)
Honor Health Research Institute (Scottsdale, AZ)	3	34
New York University (Valhalla, NY)	59	132
University of Pittsburgh Medical Center (Pittsburgh, PA)	20	13
Regional One Health (Memphis, TN)	2	9
University of Texas (UT) Southwestern, (Dallas, TX)	30	77
Virginia Commonwealth University (Richmond, VA)	1	5
