# Supplementary material for: Confirmatory Clinical Validation of a Serum-Based Biomarker Signature for Detection of Early-Stage Pancreatic Ductal Adenocarcinoma
Source: Curr Oncol. 2025 Nov 13;32(11):638. doi: 10.3390/curroncol32110638 (PMC12651218; doi:10.3390/curroncol32110638)
Supplement: Supplementary file 1 [file curroncol-32-00638-s001.zip › Table S2.pdf]

Supplemental Table 2. Analyte expression in PDAC cases and controls.

		Cases		Controls		
Analyte	Reportable Range (U/mL for CA 19-9, ng/mL for all others)	Mean	Median	Mean	Median	P-value*
TIMP1	153-1771	597.1	559	448.2	435	< 0.001
ICAM1	38-318	247.6	278	194.9	193	< 0.001
CTSD	196-771	546.6	547	404.8	373.5	< 0.001
THBS1	11817-97360	32369.3	32125	34075.5	33141	0.2915
CA 19-9	0.6-1000	208.5	78.3	19.2	11	< 0.001

* P-value comparing analyte expression between PDAC cases and controls.
