# Supplementary material for: Confirmatory Clinical Validation of a Serum-Based Biomarker Signature for Detection of Early-Stage Pancreatic Ductal Adenocarcinoma
Source: Curr Oncol. 2025 Nov 13;32(11):638. doi: 10.3390/curroncol32110638 (PMC12651218; doi:10.3390/curroncol32110638)
Supplement: Supplementary file 1 [file curroncol-32-00638-s001.zip › Table S3.pdf]

| **Supplemental Table 3. Analyte expression in males and females.** | | | |
|---|---|---|---|
| | P-values | | |
| Analyte | Full cohort | Cases | Controls |
| TIMP1 | 0.1553 | 0.356 | 0.6559 |
| ICAM1 | 0.7399 | 0.384 | 0.0511 |
| CTSD | 0.3275 | 0.3766 | 0.9103 |
| THBS1 | 0.8834 | 0.6218 | 0.8290 |
| CA 19-9 | 0.0187 | 0.0186 | 0.3937 |
