# Supplementary material for: Confirmatory Clinical Validation of a Serum-Based Biomarker Signature for Detection of Early-Stage Pancreatic Ductal Adenocarcinoma
Source: Curr Oncol. 2025 Nov 13;32(11):638. doi: 10.3390/curroncol32110638 (PMC12651218; doi:10.3390/curroncol32110638)
Supplement: Supplementary file 1 [file curroncol-32-00638-s001.zip › Table S4.pdf]

**Supplemental Table 4. Analyte expression at each collection site.**

| | | Honor Health Research Institute | New York University | University of Pittsburgh Medical Center | Regional One Health | University of Texas Southwestern | Virginia Commonwealth University |
|---|---|---|---|---|---|---|---|
| **Analyte** | **Dataset** | **Median (Range)** | | | | | |
| TIMP1 (ng/mL) | All | 448 (289 - 1018) | 429 (153 - 1633) | 510 (326 - 1771) | 540 (394 - 835) | 483 (223 - 960) | 445.5 (381 - 600) |
| | Cases | 538 (519 - 678) | 501 (235 - 1196) | 557.5 (326 - 1771) | 649.5 (464 - 835) | 634 (362 - 960) | 438 (438 - 438) |
| | Controls | 444.5 (289 - 1018) | 396 (153 - 1633) | 476 (359 - 606) | 540 (394 - 726) | 462 (223 - 776) | 446 (381 - 600) |
| ICAM1 (ng/mL) | All | 214 (92 - 318) | 188 (38 - 318) | 256 (103 - 318) | 242 (138 - 318) | 210 (84 - 318) | 195.5 (101 - 318) |
| | Cases | 239 (190 - 297) | 239 (38 - 318) | 318 (109 - 318) | 318 (318 - 318) | 287.5 (88 - 318) | 214 (214 - 214) |
| | Controls | 213 (92 - 318) | 181.5 (65 - 318) | 211 (103 - 318) | 208 (138 - 318) | 200 (84 - 318) | 190 (101 - 318) |
| CTSD (ng/mL) | All | 365 (196 - 593) | 400 (196 - 771) | 505 (214 - 771) | 377 (196 - 771) | 462 (196 - 771) | 330 (253 - 658) |
| | Cases | 413 (411 - 458) | 482 (196 - 771) | 557.5 (214 - 771) | 483.5 (196 - 771) | 629 (196 - 771) | 658 (658 - 658) |
| | Controls | 358 (196 - 593) | 366.5 (196 - 771) | 411 (302 - 771) | 377 (196 - 771) | 433 (196 - 771) | 313 (253 - 419) |
| THBS1 (ng/mL) | All | 36999 (14155 - 55836) | 28796 (11817 - 97360) | 31108 (11817 - 60819) | 19345 (11817 - 84148) | 36956 (11817 - 81476) | 36746 (19570 -44110) |
| | Cases | 43358 (14155 - 43894) | 32125 (11817 - 58533) | 27571.5 (11817 - 50651) | 13949 (11817 - 16081) | 39165 (17830 - 69094) | 37859 (37859 -37859) |
| | Controls | 36501 (14954 - 55836) | 28178.5 (11817 - 97360) | 36670 (16480 - 60819) | 36860 (11817 - 84148) | 36115 (11817 - 81476) | 35633 (19570 -44110) |
| CA 19-9 (U/mL) | All | 15.3 (0.6 - 131.5) | 14.7 (0.6 - 1000) | 36.9 (0.7 - 1000) | 15 (0.6 - 1000) | 17.1 (0.6 - 1000) | 3.2 (0.6 - 16.3) |
| | Cases | 73.1 (54.4 - 131.5) | 67.3 (0.6 - 1000) | 127 (0.7 - 1000) | 733.3 (466.6 - 1000) | 79.5 (1.8 - 1000) | 16.3 (16.3 - 16.3) |
| | Controls | 13.7 (0.6 - 95) | 11 (0.6 - 114.1) | 10.3 (3.8 - 43.6) | 5.5 (0.6 - 51.2) | 11.6 (0.6 - 1000) | 3.1 (0.6 - 6.6) |
