# Supplementary material for: Confirmatory Clinical Validation of a Serum-Based Biomarker Signature for Detection of Early-Stage Pancreatic Ductal Adenocarcinoma
Source: Curr Oncol. 2025 Nov 13;32(11):638. doi: 10.3390/curroncol32110638 (PMC12651218; doi:10.3390/curroncol32110638)
Supplement: Supplementary file 1 [file curroncol-32-00638-s001.zip › Table S5.pdf]

**Supplemental Table 5. Test performance by collection site.**								
Collection Site	Cases	Controls	TP	TN	FP	FN	Sensitivity	Specificity
Honor Health Research Institute	3	34	3	29	5	0	100%	85.3%
New York University	59	132	39	123	9	20	66.1%	93.2%
Pittsburgh Medical Center	20	13	16	11	2	4	80%	84.6%
Regional One Health	2	9	2	5	4	0	100%	55.6%
University of Texas Southwestern	30	77	28	65	12	2	93.3%	84.4%
Virginia Commonwealth University	1	5	0	4	1	1	0%	80%
