# Supplementary figures and images for: Confirmatory Clinical Validation of a Serum-Based Biomarker Signature for Detection of Early-Stage Pancreatic Ductal Adenocarcinoma
Source: Curr Oncol. 2025 Nov 13;32(11):638. doi: 10.3390/curroncol32110638 (PMC12651218; doi:10.3390/curroncol32110638)

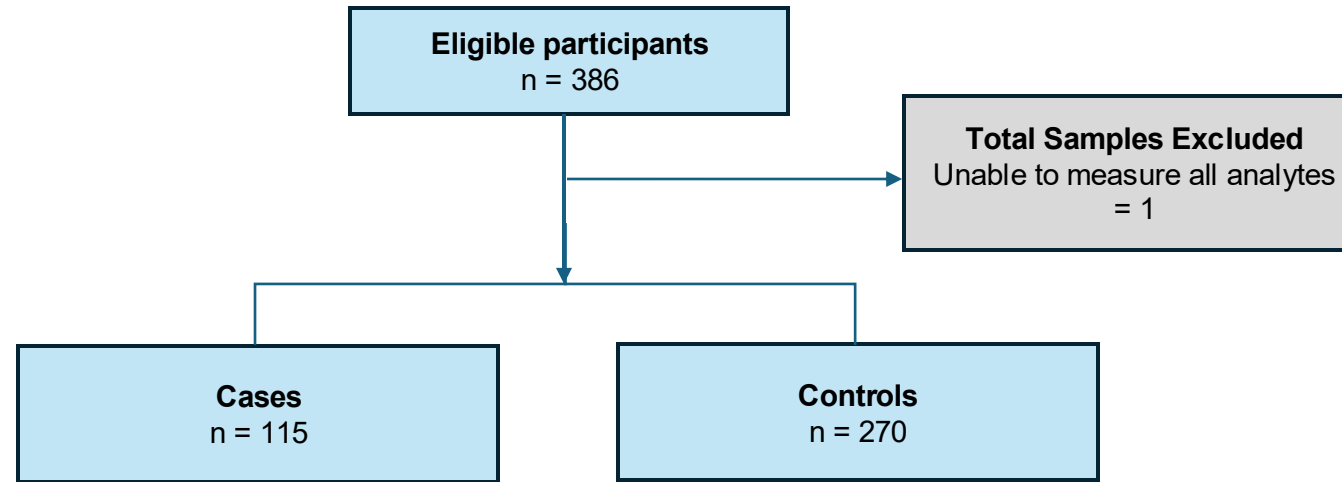

**Supplemental Figure 1. Method of sample selection.**

Supplement: Supplementary file 1 [file curroncol-32-00638-s001.zip › Figure S1.pdf]
